# Resection and Surgically Targeted Radiation Therapy for the Treatment of Larger Recurrent or Newly Diagnosed Brain Metastasis: Results From a Prospective Trial

**DOI:** 10.7759/cureus.11570

**Published:** 2020-11-19

**Authors:** Peter Nakaji, Kris Smith, Emad Youssef, Theresa Thomas, Dilini Pinnaduwage, Leland Rogers, Garrick Wallstrom, David Brachman

**Affiliations:** 1 Neurological Surgery, Barrow Neurological Institute, Phoenix, USA; 2 Neurological Surgery, Banner University Medical Center Phoenix/University of Arizona College of Medicine, Phoenix, USA; 3 Radiation Oncology, Barrow Neurological Institute, Phoenix, USA; 4 Radiation Oncology, St. Joseph's Hospital and Medical Center, Phoenix, USA; 5 Medical Physics, St. Joseph's Hospital and Medical Center, Phoenix, USA; 6 Biostatistics, Statistics and Data Corporation, Tempe, USA; 7 Radiation Oncology, GT Medical Technologies, Tempe, USA

**Keywords:** resection, metastasis, start, brachytherapy, stereotactic, srs, radiation, cs 131, collagen

## Abstract

Introduction

Achieving durable local control (LC) for larger (e.g., >2-3 cm) brain metastasis whether newly diagnosed or recurrent remains problematic. Resection (R) alone is typically insufficient and adding radiation therapy (RT) still results in a 12-month recurrence rate of 20% or more in many series. Hypothesizing that R plus immediate radiation utilizing brachytherapy may improve outcomes for this cohort of patients, we designed and prospectively evaluated a permanently implanted surgically targeted radiation therapy (STaRT) device consisting of cesium-131 (Cs-131) seeds positioned within a collagen carrier (GammaTile, GT Medical Technologies, Tempe, AZ). The device was designed to prevent direct source-to-brain contact and maintain inter-source spacing after closure.

Methods

This was a subgroup analysis of a cohort of patients with either recurrent or previously untreated brain metastases enrolled in a prospective, multi-histology single-arm trial (ClinicalTrials.gov, NCT#03088579), conducted between February 2013 and February 2018, of resection and tumor bed brachytherapy with Cs-131 containing permanently implanted collagen tiles to deliver 60 Gray (Gy) at .5 cm depth. No additional local therapy was given without progression.

Results

A total of 16 metastases in 11 patients were treated; 12 tumors were recurrent and four were previously untreated. The median preoperative maximum diameter was 3.2 cm (range: 1.9-5.1 cm). Histology was seven breasts, six lungs, and three sarcomas. The median age was 60 years (range: 41-80 years); the Karnofsky Performance Status (KPS) was 70 (range: 70-90). The cohort consisted of seven females and four males. The mean time for implantation completion was five minutes. The median overall survival (OS) was 9.3 months. At a median radiographic follow-up of 9.5 months' treatment, site progression was found in 1/16 (6%) at 10.9 months, and the median treatment site time-to-progression (TTP) has not been reached [95% confidence interval (CI): >10.9 months]. At 12 months, the Kaplan-Meier (K-M) estimates for LC after R+STaRT for all tumors was 83%; for previously untreated tumors, LC at 12 months was 100% and for recurrent tumors, it was 80%. Two tumor beds (12.5%) experienced radiation brain changes: one had grade two and the other grade three. No surgical adverse events occurred.

Conclusion

In this single-arm precommercial study, R+STaRT demonstrated excellent safety and LC in this cohort. The device has recently received FDA clearance for use in newly diagnosed and recurrent brain metastasis, and randomized clinical trials vs. standard of care treatments in both settings are scheduled to open in 2020.

## Introduction

It is estimated that 20-40% of all patients with cancer develop brain metastases and that 200,000-300,000 new brain metastases are diagnosed in the United States each year [1,2]. The size of the metastatic lesion often drives the therapeutic approach, and smaller, asymptomatic lesions in patients with known malignancies are frequently treated using radiation therapy (RT) alone and larger (e.g., >2-3 cm), symptomatic, and/or recurrent lesions in accessible locations are frequently resected [2,3]. With routinely utilized modalities, the 12-month surgical bed local control (LC) rate for smaller brain metastasis is about 90%; however, for larger lesions, the 12-month recurrence rate is 40-60% with surgery alone and reportedly 20% or more even with adjuvant RT [4-6]. This dichotomy in treatment outcomes based on size was highlighted in a recent randomized trial [4]. As patients with brain metastasis may now experience longer life expectancies owing to advances in systemic and targeted treatments, the impact of this relatively low LC rate for larger lesions means that many patients will need additional brain-tumor-directed procedures within one year of their initial treatment, with each additional procedure contributing to the potential for toxicity, diminished quality of life, and more expenditure [2,3,7].

There is a general consensus that RT is the single most effective postoperative treatment to help prevent recurrence of brain metastasis, but there is currently no consensus as to the “best” type of RT treatment in the adjuvant setting. Several topics of active debate exist regarding postoperative RT brain metastasis care, including toxicity from whole-brain coverage, optimal radiosurgery (RS) fractionation (single vs. multi-fraction), RS treatment margins, ways to deal with postoperative cavity dynamics, and the frequent exposure of external beam radiation therapy (EBRT)-transited tissues to near-tolerance doses of radiation [5-12]. These issues are all currently unresolved and likely contribute to the variable control and adverse event outcomes reported for similar tumor types and sizes [2-9].

To optimize the dosimetry and tumoricidal aspects of postoperative adjuvant radiation, a number of groups over the years have implanted radioactive sources under direct visualization at the time of the craniotomy, a technique referred to as brachytherapy [13-15]. Brachytherapy can be especially useful in the focal treatment of larger tumor beds where single-fraction stereotactic radiosurgery (SRS) or fractionated stereotactic radiosurgery (FSRS) may expose larger volumes of "innocent bystander" tissue resulting in the need to reduce the RT dose and/or the coverage area, either of which can potentially limit the effectiveness of RT in initial therapy [2,5,11,12] and recurrent disease [3,16].

A relatively new development in brachytherapy has been the commercial availability in “seed” form of the short half-life (t ½) gamma-emitting isotope cesium-131 (Cs-131) [17]. This isotope has a similar energy, and therefore treatment depth, as iodine-125 (I-125), the most commonly used radioactive isotope in intracranial and extracranial tumors. The major difference between these isotopes is that Cs-131 has a t ½ of 9.7 days compared to 59.4 days for I-125, and this markedly shorter half-life allows a much more rapid dose delivery, diminished time of radiation exposure, and possibly more rapid tumor control [18,19]. Published studies about Cs-131 to date have used a variety of doses and implantation techniques, and outcomes appear to match or exceed what is currently achievable with EBRT approaches [20-23].

Given the above-mentioned background, we hypothesized that combining maximum safe resection with the immediate initiation of radiation could improve patient outcomes in larger brain metastases. To mitigate some of the shortcomings of prior brachytherapy approaches, we designed a biocompatible device utilizing Cs-131 that prevents direct source-to-brain contact and maintains inter-source spacing after closure. This is the first prospective report about the use of this surgically targeted radiation therapy (STaRT) device in brain metastasis.

## Materials and methods

Trial design and participants

This was a subgroup analysis of the brain metastasis cohort associated with a larger prospective, single-arm, multi-histology single-institution study using a prototype medical device (ClinicalTrials.gov, NCT#03088579). The larger trial involved 108 brachytherapy procedures in 96 adult patients with aggressive intracranial neoplasms. The institutional review board (IRB)-approved informed consent was obtained from all participants. Major entry criteria included newly diagnosed or recurrent neoplasm; a planned resection; determination by the treating physicians that resection alone was unlikely to be sufficient to prevent further local recurrence; total prior same-site radiation dose of <100 Gy; capable of at least self-care (KPS of 70); and life expectancy of >six months; the trial allowed uncontrolled disease outside the operative field. Given the anticipated enrollee variability, we specified a uniform treatment of a standardized radiation prescription and implantation technique after maximum safe surgical resection. Initial results from the larger trial, the outcomes in 20 consecutive recurrent previously irradiated meningiomas, have recently been reported [22].

Technique

**Figure 1:**
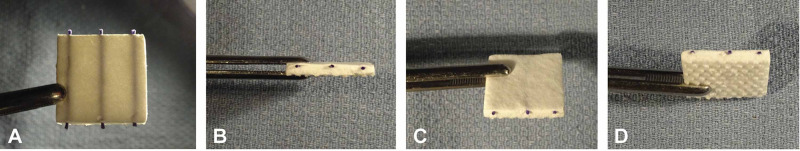
A prepared 2.5 x 2.5 x .4 cm collagen tile containing embedded Cs-131 seeds at 1-cm intervals A: the tile is shown trans-illuminated. B, C, and D are end views of the tile shown in A and depict the asymmetric source offset relative to either side of the collagen tile, with one side (C) having a relatively smooth surface, compared to the opposing side (D), which contains surface indentations (Vicryl seen as blue threads at ends of the tile; seeds are not directly visible) Cs-131: cesium-131

The technique has been previously described in detail [22]. To summarize it briefly, the expected postoperative resection bed surface in cm^2^ was estimated from a preoperative MRI, and the number of seeds required to construct the needed collagen carrier(s) with the ratio of one seed/cm^2^ of surface area was ordered in advance. The prescribed dose for all patients was 60 Gray (Gy) at a depth of .5 cm from the operative bed surface, and the design of the device in terms of seed activity and source placement within the carrier was optimized to facilitate this. During the resection in the operating room, and using a sterilized, shielded reusable stainless steel loader (GammaTile Loader, GT Medical Technologies, Tempe, AZ), the Cs-131 seeds in suture (Proxcelan, IsoRay Medical, Inc., Richmond, WA) were precisely embedded into lyophilized collagen squares (Suturable Duragen, Integra LifeSciences, Plainsboro, NJ). The Cs-containing collagen squares (referred to as “tiles” when assembled) were constructed with the radioactive sources embedded equally and symmetrically spaced at 1.0-cm intervals when considered from above or below, but with asymmetric spacing in terms of the source depth with a .3-cm offset from the bumpy side of the tile surface vs. .1 cm from the smooth side (Figure 1).

Craniotomy and maximal safe resection were performed in the usual fashion. If frozen section pathology did not confirm neoplasm, implantation was not performed. After completing resection and assuring hemostasis, the tumor bed was lined with the Cs-131-embedded collagen tiles. Tiles were typically placed against the walls of the resection bed oriented with “bumps to brain” (i.e., .3 cm from the radiation source to tile surface) to achieve the desired dosimetry. Tiles tend to remain where placed due to the hydrophilic nature of the collagen, with fibrin glue or other adhesives utilized at the surgeon’s discretion. Wound closure was accomplished in the standard fashion, with the replacement of native cranium in all cases. The operating room was surveyed throughout the procedure to ensure staff radiation exposure was in compliance with applicable statutes [24].

Postoperative Care

All patients received routine postoperative care and written discharge instructions appropriate for the surgical procedure and radioactivity levels present at the time of hospital discharge [24]. No additional local therapy was given without progression.

Follow-up

**Figure 2:**
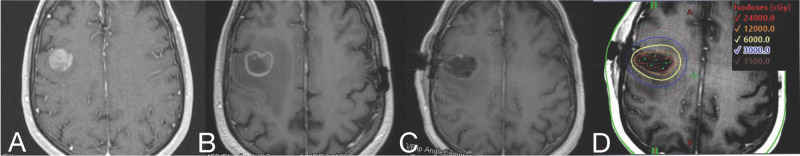
Example of a trial case (case 6, Table 2) A: MRI at initial SRS treatment; B: MRI at recurrence; C: initial postoperative scan with devices implanted; D: post-implant dosimetry with selected isodose lines; doses are in Gy. All scans are axial T1 post-contrast MRI: magnetic resonance imaging; SRS: stereotactic radiosurgery

Postoperative MRI and stereotactic, noncontrast, thin-cut CT scans were obtained within three days for all patients, and the extent of surgical resection (subtotal resection vs. gross total resection) was determined from these exams. Post-implant dosimetry was calculated using commercially available treatment planning software (BrachyVision, Varian, Palo Alto, CA). An example case is shown in Figure 2.

Follow-up visits and MRIs varied according to clinical needs but were typically scheduled every three months for the first year and every four to six months thereafter. Local failure was defined as new or progressive enhancement within 10 mm of the operative cavity at any time during follow-up, a positive biopsy, or the delivery of additional same-site local therapy.

Clinical outcome measures

Outcomes investigated included treatment site LC (including overall LC by size and by prior treatment), treatment site time-to-progression (TTP), treatment site TTP after the most recent prior same-site treatment (for previously treated tumors), overall survival (OS), distant brain failures (DBF), number and type of subsequent therapies, and radiation and surgical adverse events using Common Terminology Criterion for Adverse Events (CTCAE) Version 5.0. [25].

Statistical analysis

TTP was determined using the standard Cox proportional-hazards model and was used to assess the effect of variables on TTP. Survival estimates of TTP were given, with 95% confidence intervals, where appropriate. We also used lognormal frailty models to control for the effect of the individual patient and individual tumor on TTP [26]. Analyses were conducted using SAS 9.4 (SAS Institute Inc., Cary, NC).

The number of months was calculated as the number of days divided by 30.4. For analyses at the site level, standard errors and confidence intervals were not adjusted for multiple sites per subject. We had one subject with four sites, one subject with three sites, and nine subjects with one site each. OS was calculated based on the date of the initial implant.

## Results

Patient demographics and characteristics

Group demographics and clinical information are presented in Table 1, and the individual patient-level outcomes are summarized in Table 2.

**Table 1:** Characteristics of 16 tumors in 11 patients treated with resection+STaRT Note: all values except patient sex and age are given on a per-case (vs. per-patient) basis. Continuous variables are given as median (range). Proportions are given as fractions (percentage) STaRT: surgically targeted radiation therapy; Cs-131: cesium-131; mCi: milliCurie

Variables	Values
Sex	
Female	7/11 (64%)
Male	4/11 (36%)
Age at initial diagnosis, years	58 (41-75)
Age at resection with Cs-131 tile implantation, years	60 (41-80)
Lesion location	
Parietal	7/16 (44%)
Frontal	4/16 (25%)
Temporal	3/16 (19%)
Posterior fossa	2/16 (12%)
Treatment status at the time of implant	
No prior local treatment	4/16 (25%)
Prior local treatment	12/16 (75%)
If prior local treatment, types	
Resections	1.5 (0-3)
Radiation courses	1.5 (1-2)
Time to progression after prior treatment, months	4.8 (1.9-22)
Preoperative maximum tumor diameter, cm	3.1 (1.9-5.1)
The extent of resection at Cs-131 tile placement	
Gross total	16/16 (100%)
Histology	
Breast	7/16 (44%)
Non-small cell lung cancer	5/16 (31%)
Sarcoma	3/16 (19%)
Small cell lung cancer	1/16 (6%)
Karnofsky Performance Status	70 (70-90)
Cs-131 seeds implanted, No.	18.5 (5-63)
Radioactivity implanted, mCi	71.3 (18.5-227.4)
Median observation period, months	9.3 (1.4-28)
Median radiographic follow-up, months	9.5 (<1-25.2)

**Table 2:** Characteristics of individual patients, grouped by prior same-site treatment history A: alive; BT: brachytherapy; D: dead; DBF: distant brain failure (>1 cm from tumor bed); Dia: diameter; Dx; diagnosis; EBRT: external beam radiation therapy; GK: Gamma Knife; KPS: Karnofsky Performance Status; LITT: laser interstitial thermal therapy; LP: local progression of the disease (<1 cm from tumor bed); N: no; N/A: not applicable; No.; number; NSCCA: non-small cell carcinoma; Pt: patient; R: resection; RT: radiation therapy; SRS: stereotactic radiosurgery; TTP Pre: time to progression after most recent prior treatment; TTP Post: time to progression after implantation of Cs tiles; Tx: treatment; Vol: volume of the tumor (preoperative); WBRT: whole-brain radiotherapy; Y: yes *If patients had more than one site treated on the study, they are listed as 1b, 1c, etc. ^Prior same-site R/LITT/SRS/EBRT (No.) refers to the type and number of each prior same-site procedure; if none, a “0” is used ^†^Prior RT doses at implant site are given as total Gy, without modification for treatment type (e.g., 60 Gy external beam RT, followed by 17 Gy SRS is given as 77 Gy) ^‡^Where no local progression occurred, value is given as "--"

Case*	Prior same- site Tx	Sex/age (years)	KPS	Dx	Prior same-site R/LITT/SRS/EBRT (No.)^^^	Prior same-site RT dose (Gy)^†^	Most recent prior same-site Tx	Max preop dia (cm)	MRI stability post (mo)	LP	TTP pre/post (mo)^‡^	Implant site RT brain injury Y/N	Additional brain Rx			OS (mo)/status	Cause of death
													Y/N	Reason	Type		
1a	Y	M/74	70	Sarcoma	2/1/1/1	77	R	3.4	10.9	Y	3.4/10.9	N	Y	LP, DBF	GK to tumor bed; GK, BT to DBF	21.3/D	Systemic progression
1b	Y	M/74	70	Sarcoma	2/1/1/1	77	LITT	3.1	13.3	N	2.1/--	N	As per 1a				
1c	Y	M/74	70	Sarcoma	3/1/1/1	77	R	3.8	8.4	N	4.6/--	N	As per 1a				
2	Y	F/64	80	Breast	0/0/1/0	16	SRS (GK)	4.3	16.3	N	25.8/--	N	Y	DBF	GK, LITT	20.0/D	Systemic progression
3a	Y	F/53	70	Breast	2/0/1/0	15	SRS (GK)	5.1	25.1	N	1.0/--	Y	Y	DBF	GK, BT	28.0/D	Systemic progression
4	Y	F/41	70	Breast	0/0/2/0	32	SRS (GK)	2.0	0.03	N	17.2/--	N	N	N/A	N/A	3.9/D	Systemic progression
5	Y	M/66	70	NSCCA lung	1/0/0/1	30	R	3.7	0.1	N	2.7/--	N	N	N/A	N/A	1.7/D	Cardiac arrest
6	Y	M/60	90	NSCCA lung	0/0/1//0	16	SRS (GK)	2.4	5.9	N	5.1/--	N	Y	DBF	GK, WBRT	7.2/D	Systemic progression
7	Y	M/79	90	NSCCA lung	1/0/2/0	33	R+SRS (GK)	3.6	4.34	N	31.2/--	N	Y	DBF	GK	5.6/D	Remote intracranial progression
8	Y	F/49	80	NSCCA lung	3/0/1/0	17	SRS (GK)	2.1	21.1	N	7.8/--	Y	Y	DBF	BT	25.2/A	Alive
9	Y	F/46	70	Small cell lung	1/0/0/2	55	R	3.2	4.3	N	1.9/--	N	N	N/A	N/A	12.0/D	Systemic progression
3b	Y	F/54	70	Breast	1/0/0/0	N/A	R	3.1	10.6	N	15.3/--	N	As per 3a				
3c	N	F/54	70	Breast	N/A	N/A	N/A	1.9	21.4	N	N/A	N	As per 3a				
3d	N	F/54	70	Breast	N/A	N/A	N/A	3.0	10.6	N	N/A	N	As per 3a				
10	N	F/80	70	Breast	N/A	N/A	N/A	2.8	0.03	N	N/A	N	N	N/A	N/A	3.3/D	Systemic progression
11	N	F/57	70	NSCCA lung	N/A	N/A	N/A	3.6	0.03	N	N/A	N	N	N/A	N/A	1.4/D	Post-discharge hip fracture, hospice

From February 2013 to February 2018, 16 separate metastatic lesions in 11 patients were treated on the trial. The median maximum preoperative diameter was 3.2 cm (range 1.9-5.1 cm). At the time of enrollment, 12 treatment sites (75%) were recurrent after prior treatment, and four sites (25%) were previously untreated. For previously treated patients, prior same-site resections were none in three patients, one in four patients, two in three patients, and three in two patients (Table 2). Prior same-site RT courses were none in one patient, one in five patients, and two in six patients; the median prior same-site RT dose was 32 Gy (range: 15-110 Gy) (individual dose and modality details are provided in Table 2). The median age at implant was 60 years, and the median KPS was 70 (range 70-90). The cohort consisted of seven females and four males. Tumor types treated were breast (seven), non-small cell lung (five), sarcoma (three), and small cell lung cancer (one). Lesion location was recorded as parietal in six, frontal in four, temporal in three, posterior fossa in two, and parietal-occipital in one. Gross total resection was accomplished in all patients (Table 2).

The median observation period for all patients was 9.3 months (range: 1.4-28 months), and median radiographic follow-up after tile placement was 9.5 months (range: <1-25.2 months) (Table 1). Two patients expired <90 days from surgery, one from cardiac arrest and the other after pathological hip fracture and subsequent hospice referral (Table 2; cases 5 and 11, respectively). Excluding these early deaths, the median OS was 12.0 months (range: 3.3-28 months) (Table 2). No patient was lost to follow-up.

The mean number of seed sources used was 19 (range: 5-63), and the mean activity implanted was 71 mCi (range: 18.5-227 mCi) (Table 1). The added time to surgical resection for tile placement ranged from two to 22 minutes (median: seven minutes) and dropped during the course of the trial to under five minutes.

Local control

**Figure 3:**
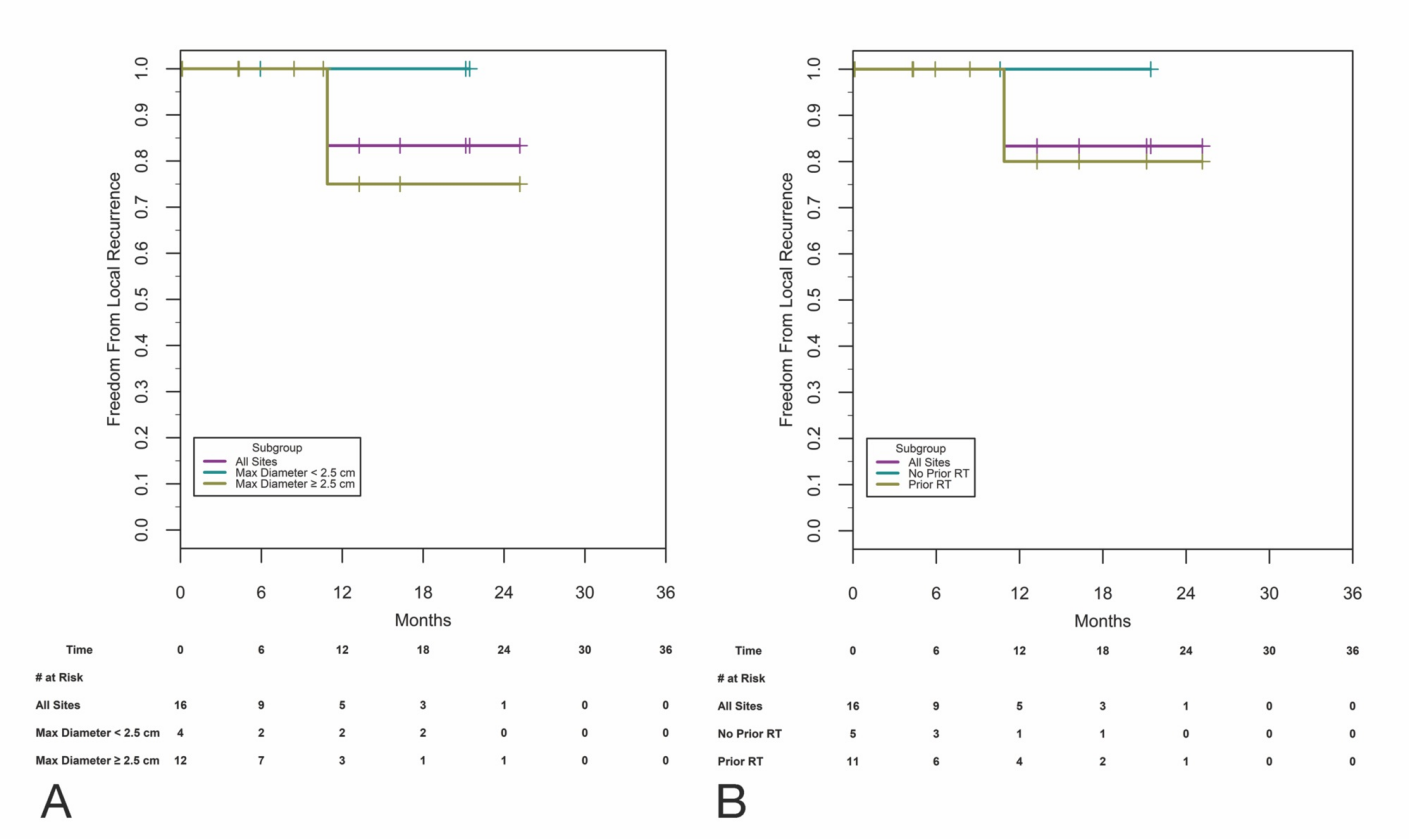
Local control by maximum preoperative diameter and by radiation treatment status A: local control by maximum preoperative enhancing diameter. LC for all tumors, tumors of <2.5 cm, and tumors of >2.5 cm are shown. B: LC by radiation treatment status. Numbers of resection beds at risk at specified time points are shown below the graphs RT: radiation therapy; LC: local control

**Figure 4:**
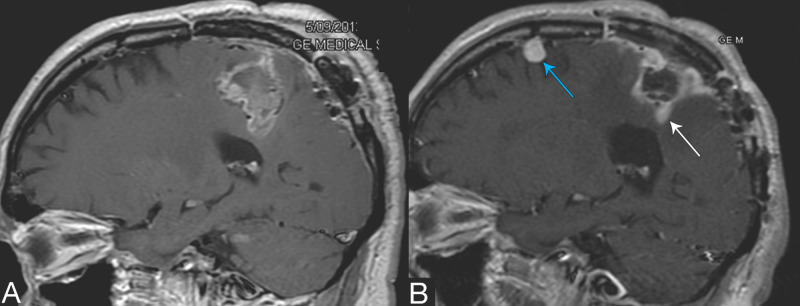
SRS salvage treatment in the lone patient with treatment failure A: initial postoperative scan with Cs tile devices implanted after the resection of 3.4-cm sarcoma metastasis; B: the image on the day of SRS treatment 10.9 months after R+Cs tile implant and shows one of the four metastasis treated (blue arrow) and the site of treatment to an area of persistent nodular enhancement (white arrow). Other new metastatic sites are not shown. The patient expired of systemic disease at 23 months. Scans are sagittal T1 post-contrast SRS: stereotactic radiosurgery

By maximum preoperative diameter, the Kaplan-Meier (K-M) estimated LC at 12 months for all tumors, tumors of <2.5 cm, and those of >2.5 cm was 83%, 100%, and 75%, respectively (Figure 3A). Treatment site progression occurred within one implanted area (1/16, 6%) at 10.9 months (Figure 4). Following treatment, the median treatment site TTP for the group has not been reached (95% CI gives a lower limit of at least 10.9 months).

For tumors with no prior same-site treatments, no treatment site failures were encountered, and the K-M estimated LC at one year was 100% (Figure 3B). For tumors with previous same-site treatments, 1/12 (8%) failed at the treatment site, and the estimated LC at one year was 80% (Figure 3B).

Time to treatment site progression after R+STaRT vs. time to treatment site progression after most recent prior treatment: for tumors with previous same-site treatments (n = 12), we examined the treatment site TTP for each tumor after trial therapy and compared this to treatment site TTP after the most recent prior treatment. The median treatment site TTP for the prior treatment was 4.8 months (95% CI: 1.9-22.0 months) and the median time after R+STaRT has not been reached (95% CI gives a lower limit of at least 10.9 months). Treatment site LC at 12 months with the prior treatment was 33% vs. 80% after R+STaRT (SE: 13.61 and 17.89, respectively). With site-level frailty term, hazard ratio (HR) was 0.052 (p = 0.0073; 95% CI: 0.006-0.452). With subject-level frailty term, HR was 0.061 (p = 0.0095; 95% CI: 0.007-0.504). With no frailty term, HR was 0.082 (p = 0.0175; 95% CI: 0.010-0.645). In single-variable models, age, gender, number of prior surgeries to index site, number of prior SRS events to index site, and number of prior systemic to index site were not significant at the 0.05 significance level.

Overall survival

At a median observation period of 9.3 months (range: 1.4-28 months), five patients (45%) remained alive. The median survival was estimated at 9.3 months (95% CI: 1.7-21.3 months). OS was calculated on a per-patient basis, based on the date of the initial implant.

Toxicity

No patient remained hospitalized beyond what was typical for the surgical procedure. Two patients expired <90 days, one from cardiac arrest and one after a pathological hip fracture (Table 2; cases 5 and 11). Two implant sites (2/16, 12%) exhibited radiation brain injury (RBI) after implantation and fully resolved with two- and four-week courses of dexamethasone, respectively (Table 2, cases 3a and 8). Both were in patients who had received same-site RT prior to the implantation. No deaths were related to the reoccurrence of a treated lesion (Table 2), and no staff or caregiver toxicities occurred.

Subsequent brain therapies

After the implantation, six patients experienced intracranial failure. Five patients experienced only DBF and all underwent additional brain treatments, with whole-brain radiotherapy (WBRT) used in one patient and four patients receiving a total of nine site-specific treatments: SRS in five cases, on-trial brachytherapy in three, and laser interstitial thermal therapy (LITT) in one (Table 2). The one patient who experienced both DBF and implant bed failure underwent salvage SRS for both (Table 2, Figure 4).

Radiation safety

All radiation exposure readings during implant construction, operating room use, and discharge were at acceptable levels per statute [24]. At the end of the cranial closure, all patients had exposure levels below 6 mR/hr, the typical level specified for home discharge [24]. Upon discharge, patients were given instructions related to their recorded radiation level and living situation, to minimize radiation exposure to family members/caregivers.

## Discussion

In this report, we presented our experience with surgery plus a prototype implantable radiation device in the treatment of patients with larger recurrent or newly diagnosed brain metastasis enrolled in a prospective single-arm observational trial. The implant utilized was a novel biocompatible device that holds multiple radiation sources while also functioning as a three-dimensional source spacer.

The path to an optimum outcome for patients with larger metastasis is not well defined either in the initial or recurrent setting. The current National Comprehensive Cancer Network (NCCN) guidelines note that surgery is “preferred” for newly diagnosed limited disease patients, and include resection as an option in most other scenarios, including for the setting of recurrent brain metastasis in patients with “limited” or “extensive” disease [27]. These guidelines also include references to repeat irradiation if “further RT possible,” as repeat RT use is often limited by toxicity concerns, not lack of efficacy [27].

Some of the known shortcomings of adjuvant EBRT in either initial or recurrent brain metastasis settings include 1) an increasing injury risk as the irradiated brain volume increases; 2) tumor regrowth during the time needed for initial wound-healing prior to starting EBRT; and 3) a difficulty on postoperative imaging in precisely determining the area(s) needing treatment [9-12,16]. While post-resection brachytherapy with low-energy sources can potentially treat a smaller volume of normal tissue than EBRT (including SRS) and also allow the initiation of treatment immediately, it is not without drawbacks and has not achieved routine use status in brain tumors [13,27]. Previously published brachytherapy series have pointed out several problems with existing implant techniques and devices including a) the potential for tissue injury from high radiation doses arising from direct tissue contact with either surface placement or intra-parenchymal insertion of sources; b) determining and maintaining the precise inter-seed operative bed geometry needed for accurate dose delivery; and c) surgical team and caregiver concerns regarding radiation exposure [13-15,18,20-22].

Local control

LC in brain metastasis is often benchmarked to one year. Our LC of 83% at one year for this group of very challenging patients was encouraging (Figures 2, 3), particularly as achieving freedom from local progression is more difficult when treating larger and/or recurrent disease [3-6,8,9,16]. Our one-year LC was similar to the findings from Brown et al. of 80% for resection and adjuvant WBRT in previously untreated patients [6]. While the use of adjuvant WBRT eliminates concerns over precisely where to treat, this tradeoff is increasingly unpopular clinically [2,6,7]. We and other centers investigating brain brachytherapy for metastasis believe that it may be particularly useful in larger lesions as control appears relatively independent of size [20-23]; our study results support this finding. Figure 2 shows our LC by size grouping and with only one failure. Interestingly, this failure was in a patient with a 3.4-cm sarcoma metastasis who developed four new intracranial lesions >10 months post-implant. At the time of SRS to these new lesions, a small area of persistent enhancement in the implant area was also treated with SRS, and this was scored as a failure; he died of systemic spread at 23 months (Figure 4; Table 2, case 1a).

Immediacy

Unlike EBRT therapies, brachytherapy allows adjuvant treatment to start immediately. To take full advantage of the minimum tumor burden, we chose to use the 9.7-day t ½ Cs-131 as the shorter t ½ of Cs-131 delivers 50% of the therapeutic dose within <10 days of surgery and 88% of the dose within 30 days vs. 60 days for 50% delivery and 300 days for 88% dose with the more commonly used isotope I-125 [19]. This markedly shorter time for dose delivery has been postulated to offer a significant advantage in the treatment of tumors exhibiting a relatively short doubling time [19].

Safety

Historically, brain brachytherapy literature has been associated with high rates of brain necrosis and/or wound complications [13]. Despite the heavily pretreated nature of many of our patients, the adverse event rate was at or below what was expected. No wound complications related to the implant procedure were seen (Table 2). Two of the 16 treatment areas developed evidence of radiation brain changes, both at sites that had previously undergone SRS, and neither patient required long-term steroids (Table 2). At similar follow-up times, findings in published literature related to traditional brachytherapy for radiation brain injury complications range as high as 24-60%; SRS in re-irradiated patients report 11-16% necrosis rates, and surgical complications in neoplasm resections have been reported to be between 1-24% for dural closure-related complications and .5-4% for wound infections, respectively [3,12,13,16,20,22,28]. One explanation for the low brain injury rate observed in our experience vs. prior brachytherapy publications may be attributed to the difference in implant technique. Prior brachytherapy series have often positioned the seed sources directly on or within the substance of the brain, resulting in extremely high tissue doses at the point of contact, potentially exceeding tolerance [13-15,22]. In contrast, the collagen device used in this trial, when whetted by cerebrospinal fluid, was specifically designed to offset the sources 3 mm from the brain. Even though this is a relatively small distance, by leveraging the physics principle known as the inverse square law, the design of the device lowers the resection bed surface dose to ~120-150 Gy, falling rapidly to ~60 Gy at a depth of .5 cm [22]. Additionally, the use of the collagen tiles provided fixed inter-source spacing during placement and minimized post-closure source shifting, lowering the risk of areas of localized overdose (“hot spots”) from source clumping or areas of underdose (“cold spots”) that could lead to tumor recurrence [22]. We believe these aspects of the tile design allowed a more predictable and uniform radiation dose than had been previously achievable in brain brachytherapy and that this design may have contributed to the lessening of necrosis and wound breakdown. Also, in previously irradiated tumors, confirming recurrence with intraoperative pathology prior to implantation is part of the workflow (see Materials and Methods). Thus, the likelihood of adding additional radiation to symptomatic radiation injury is almost eliminated. One additional possibility is the treatment prescription dose, 60 Gy at .5 cm, which was relatively “conservative” in comparison to other brain brachytherapy series [13-15,20,21]. The specification of this dose was a deliberate attempt to lessen the necrosis risk given our heavily pretreated patient cohort. Nevertheless, this dose seems to have been sufficient for tumor control (Figure 3).

Potential study limitations

Our data were drawn from a subset cohort that was part of a larger, single-arm, prospective observational trial. The smaller number of patients involved, the lack of a control group, and the single-site nature of the trial present several challenges for data interpretation. This proof of concept trial was deliberately designed to accrue quickly by enrolling patients with varied metastatic histologies, controlled and uncontrolled disease outside of the operative field, and varied prior treatments. As reported in detail in Table 2, most patients had recurrent disease and had been heavily pretreated.

Despite this being an inherently unfavorable cohort, the median OS for all patients was 9.3 months, and we believe this survival was probably sufficient to judge the harms and LC benefits. For assessment of surgical harms, typically judged within 90 days, the reported follow-up appears sufficient [28]. In terms of radiation brain changes, the answer is also that it was probably sufficient [13]. A recent meta-analysis of harms in brain brachytherapy re-irradiation used a minimum of six months of follow-up post-second treatment for study inclusion, and our median follow-up was 9.5 months. LC assessment was also likely long enough in that that the median TTP after R+STaRT has not been reached vs. the median treatment site TTP for the prior received treatments of 4.8 months (Table 2).

One question that arises is whether the durable LC was due to surgery alone. For any one case, this is a possibility, but we feel this is generally unlikely for multiple reasons. First, the majority of enrolled patients had progressed despite both prior same-site surgery and prior adjuvant radiotherapy (Table 1). Secondly, as almost all patients received their prior care at the study institution and also progressed quickly after their prior treatment (mean of 4.8 months), the argument for improved surgical expertise as the cause of the improved outcomes seems highly unlikely (Table 1). In addition, as discussed above, the literature suggests that even with excellent surgical care, resection alone frequently results in local recurrence in larger brain metastasis [1,5].

Another possibility is that the patients’ implant site LC rate was positively impacted by subsequent treatment. We believe that any impact of these treatments on the implant site control was likely small: there was only one instance of adjuvant immunotherapy and one of WBRT, and both were in the same person with the latter used when the former had failed (case 6, Table 2).

One additional shortcoming that may be raised is that we did not report the radiation biologically effective dose (BED) from this treatment. During the drafting of the protocol and again during manuscript preparation, we examined this subject in detail but ultimately concluded that BED modeling in brain tumor brachytherapy had not achieved a clear consensus. For this reason, we decided that the inclusion of a BED “number” without a full and open examination of the various current opinions and underlying mathematical models was not a wise choice and that a proper treatment of the subject was beyond the scope of this initial clinical paper. We are hopeful that in the future, brain brachytherapy BED calculations will be a useful tool, and could help place treatment outcomes in perspective with the modalities of SRS and FSRS.

## Conclusions

We have presented our results about utilizing a novel intraoperative device designed to facilitate STaRT. In the presented patient cohort, many of whom were heavily pretreated, resection plus the use of this brachytherapy device demonstrated excellent tumor bed control with minimal side effects. No single factor seems to account for the entirety of the safety or efficacy outcomes achieved, and we believe a comprehensive explanation likely includes a) immediacy of treatment initiation, b) dose delivery at the time point of least residual tumor, c) dose accuracy, d) specified dose, and e) carrier design aspects discussed above. Given the reported outcomes, treatment utilizing this device presents a potentially effective therapeutic option for those with large and/or recurrent brain metastases, a category of tumors that currently experience relatively poor LC with existing treatment options. A commercially-produced version of the device has recently received the FDA clearance for use in both newly diagnosed and recurrent brain metastasis. To validate these outcomes, randomized clinical trials in both newly diagnosed brain metastasis (NCT 04365374, Clinicaltrials.gov) and in previously irradiated metastasis vs. standard of care treatments are opening in 2020.
